# Mortality and associated factors in post-stem cell transplant patients: a two-year cohort in a public hospital in Peru

**DOI:** 10.17843/rpmesp.2025.423.14389

**Published:** 2025-09-02

**Authors:** Mario Agramonte-Vilca, Karol Moscol-Chavez, Lourdes Aranda-Gomero, Alfredo Wong-Chang, Stalin Vilcarromero, Cesar Copaja-Corzo

**Affiliations:** 1 Universidad San Antonio Abad del Cusco, Cusco, Peru. Universidad Nacional San Antonio Abad del Cusco Universidad San Antonio Abad del Cusco Cusco Peru; 2 Servicio de Hematología Especializada; Unidad de Trasplante de Progenitores Hematopoyéticos; Hospital Nacional Edgardo Rebagliati Martins, EsSalud Lima, Peru. Servicio de Hematología Especializada Unidad de Trasplante de Progenitores Hematopoyéticos Hospital Nacional Edgardo Rebagliati Martins, EsSalud Lima Peru; 3 Servicio de Infectología, Hospital Nacional Edgardo Rebagliati Martins, EsSalud, Lima, Peru. Servicio de Infectología Hospital Nacional Edgardo Rebagliati Martins EsSalud Lima Peru; 4 Unidad de Investigación para la Generación y Síntesis de Evidencias en Salud, Universidad San Ignacio de Loyola, Lima, Peru. Universidad San Ignacio de Loyola Unidad de Investigación para la Generación y Síntesis de Evidencias en Salud Universidad San Ignacio de Loyola Lima Peru

**Keywords:** Opportunistic Infections, Hematopoietic Stem Cell Transplantation, Graft vs Host Disease, Sepsis, Leukemia, Hospital Mortality

## Abstract

**Objective.:**

To determine mortality and associated factors in patients who received an allogeneic hematopoietic stem cell transplant at the Hospital Nacional Edgardo Rebagliati Martins, between January 2017 and December 2022.

**Materials and methods.:**

A retrospective cohort study was conducted on patients who underwent allogeneic hematopoietic stem cell transplantation (HSCT) between 2017 and 2022. Death at one hundred days and two years, and its associated factors, were evaluated. Proportional Cox regression models were used.

**Results.:**

342 post-HSCT patients were included, with a median age of 23 years (interquartile range: 10 to 39), of whom 53.5% were women. The most frequent diagnosis for the transplant was acute lymphoblastic leukemia at 54.1%. Mortality in the first 100 days post-HSCT was 8.2%, and at two years was 30.4%. In the multivariate regression at one hundred days, factors associated with a higher risk of mortality were age over 50 years (HRa: 6.97; 95% CI: 1.18−41.23), being a recipient of a haploidentical transplant (HRa: 3.57; 95% CI: 1.13−11.24), and sepsis as a complication (HRa: 68.78; 95% CI: 19.32−244.84). In the two-year analysis, acute myeloid leukemia (1.72; IC 95%: 1.08-2.74), haploidentical transplant (HRa:1.81; 95%CI: 1.21-2.72), and disease relapse (HRa: 4.17; 95%CI%: 2.75-6.31) were associated with a higher risk of death.

**Conclusions.:**

The mortality found was low and similar to that of countries with higher incomes; however, it is necessary to formulate interventions that reduce the incidence of modifiable factors such as sepsis.

## INTRODUCTION

Hematopoietic stem cell transplantation (HSCT), which consists of the infusion of pluripotent stem cells into individuals previously conditioned with antitumor radiochemotherapy, is a vital therapeutic intervention for various malignant and non-malignant hematological pathologies, and even for some primary immunodeficiencies ^1^. HSCT can cure and/or prolong the life of onco-hematological patients and is an increasingly common procedure in various regions of the world ^1^^-^^3^. This is due to the inclusion of older patients, less toxic chemotherapy conditioning regimens, a greater number of unrelated donors, advances in high-resolution human leukocyte antigen (HLA) typing, and the use of new immunosuppressive drugs ^1^^,^^4^.

However, HSCT also carries risks and is not without complications ^1^. These include the risk of infections due to induced immunosuppression (radiochemotherapy prior to transplantation), the risk of toxicity secondary to chemotherapeutic agents, graft-versus-host disease (GVHD), graft failure, and malignancy recurrence ^1^^,^^4^. Importantly, the Center for International Blood and Marrow Transplant Research (CIBMTR) in the United States reported that between 2016 and 2021, in the first 100 days of post-HSCT follow-up, infections, organ failure, and underlying disease relapse contributed to mortality in 25%, 24%, and 23% of cases, respectively ^5^. Bacterial infections are more frequent in the early post-transplant period, while the reactivation of latent viral forms usually occurs in later phases ^6^^,^^7^. Furthermore, moderate to severe acute GVHD has a synergistic effect on cytomegalovirus (CMV) reactivation and has been associated with a decline in the quality of life of survivors ^2^^,^^8^.

In Peru, HSCT has been performed since 1994, and although the number of transplants has greatly increased over the years ^9^, research in Latin America, and especially in Peru, has been limited. The hematopoietic stem cell transplant unit (HSCTU) of the Hospital Nacional Edgardo Rebagliati Martins (HNERM) is the largest in Peru, making it a national reference center. Given the relevance of the topic and the lack of information, analyzing the data from the HSCTU of the HNERM will provide an initial overview of transplants in Peru that can generate information for decision-making. In this sense, this study aims to determine the mortality and associated factors in patients receiving allogeneic hematopoietic stem cell transplants at the HNERM, between January 2017 and December 2022.

**KEY MESSAGES**

Motivation for the study. In Peru, the number of patients treated with hematopoietic stem cell transplantation (HSCT) has increased, but information on post-HSCT outcomes is limited or non-existent.

Main findings. Between 2017 and 2022, 744 HSCTs were performed at the Hospital Nacional Edgardo Rebagliati Martins, of which 394 were allogeneic. Mortality at 100 days post-HSCT was 8.2% and at two years, 30.4%; these figures were low and comparable to those reported in higher-income countries. The main factors associated with mortality were sepsis and disease relapse.

Implications. Greater investment and the implementation of more effective public health strategies are necessary to continue increasing the number of transplants and improve long-term survival.

## MATERIALS AND METHODS

### Study design and setting

A retrospective cohort study was conducted by reviewing the clinical records of patients treated at the HSCTU of the HNERM in Peru. The manuscript was prepared following the Strengthening the Reporting of Observational Studies in Epidemiology (STROBE) guidelines for reporting observational studies ^10^.

In Peru, there are 10 hematopoietic stem cell transplant centers accredited by the Ministry of Health (MINSA). Seven of them are located in the Peruvian capital and three in the provinces. The HNERM is one of the four centers accredited for allogeneic transplantation ^11^, performing transplants for onco-hematological diseases and other non-oncological diagnoses, such as aplastic anemia or primary immunodeficiencies. The HSCTU has performed more than 1600 transplants since 1994, including autologous and allogeneic (identical or non-identical family member, the latter also called haploidentical), and is a specialized national reference unit ^12^^-^^14^.

### Population

All patients who received an allogeneic transplant between January 2017 and May 2022, of all ages, were included. In addition, the patient’s follow-up had to be longer than 730 days (two years) and have the final follow-up outcome recorded (survived or did not survive). Clinical records of patients who did not complete this follow-up at the time of the study were excluded. Data from all patients who met the inclusion criteria were analyzed.

### Procedures and techniques

After obtaining approval from the ethics committee and authorization from the clinical management of HNERM, access was granted to the HSCTU patient registry. This is a digital Excel registry created from the medical follow-up of patients (pre- and post-transplant period) and maintained by specialist hematology physicians of the HSCTU. The database was managed in Microsoft Excel. To ensure data integrity and accuracy, the principal investigator downloaded the database and performed a double-check by comparing the information recorded in the database with the electronic health records. Systematic data cleaning was implemented to detect and correct errors, such as outliers (e.g., hemoglobin of 87 g/dL), which were validated and adjusted according to the original electronic health record. For missing data, an exhaustive search of the electronic health records was conducted; if a piece of data could not be located, it was left blank and analyzed as missing data, without imputation. Finally, to ensure confidentiality, all personal information was removed and random study codes were assigned before proceeding with the statistical analysis.

At HNERM, autologous and allogeneic hematopoietic stem cell transplants from related donors are performed: with identical HLA compatibility (100% HLA match) and haploidentical (50% of HLAs), in addition to the identification of donor-specific antibodies, as appropriate. All patients receiving HSCT undergo studies such as echocardiogram, spirometry, and dental and otorhinolaryngological de-focalization. In addition, a complete serology study for toxoplasma, human herpesviruses, syphilis, HIV, hepatitis B and C, biochemical analysis, and evaluation of venous access is performed. Patients with onco-hematological disease must be in complete remission of the disease before undergoing HSCT.

Regarding the transplant procedure: the conditioning treatment, which is the chemotherapy regimen with or without radiotherapy administered before the infusion of hematopoietic stem cells, can have a myeloablative, non-myeloablative, or reduced-intensity intention depending on the combination and dosage of antineoplastics and immunosuppressants. The commonly used regimens are, for example: cyclophosphamide 50 mg/kg/day and TBI 1200 cGy (centigray) as myeloablative, and fludarabine 30 mg/m2/day, busulfan 0.8mg/kg q/6h (for 12 doses) as reduced or intermediate intensity. In parallel, donors receive colony-stimulating factor (CSF 10 mcg/kg) starting four days before the stem cell infusion, to be subsequently collected. The source of hematopoietic stem cells used is peripheral blood and it is collected by leukapheresis. For GVHD prophylaxis, compatible related recipients (8/8-10/10 HLA antigens) receive cyclosporine 5 mg/kg/day from day -1 post-HSCT, and then on day +1 post-HSCT, methotrexate 15 mg/m2, followed by methotrexate 10mg/m2 on days +3, +6, and +11 post-HSCT. In turn, haploidentical transplant recipients (4/8 HLA antigens) receive cyclophosphamide 50 mg/kg/day on day +3 and +4 post-HSCT, and tacrolimus 0.075 mg/kg, associated with mycophenolate 15 mg/kg/dose starting from day +5 post-HSCT. To prevent viral infections, acyclovir is administered.

### Variables

*Outcome variable*

Late mortality was considered when death occurred after HSCT up to 730 days of follow-up (two years). Early mortality was considered as death within the first 100 days of follow-up after the transplant ^15^. To record mortality, the digital clinical records of EsSalud (Social Health Insurance) patients, the electronic file of the National Death Information System (SINADEF) ^16^, and the National Registry of Identification and Civil Status (RENIEC) of Peru ^17^ were evaluated. Although web access to both sources is public, the verification of deaths is restricted (only authorized personnel can access the information). In our case, the access request was managed through administrative procedures with EsSalud.

*Exposure variables*

The exposure variables were grouped into: 1) clinical characteristics: age (≤11/12-17/18-49/≥50), sex (male/female), hematological diagnosis (acute lymphoblastic leukemia/acute myeloid leukemia/severe aplastic anemia/myelodysplastic syndrome/Hodgkin and non-Hodgkin lymphoma/others); due to the small number of observations for the regression analysis, it was decided to recategorize this variable (acute lymphoblastic leukemia/acute myeloid leukemia/others), time from diagnosis to transplant (≤1 year/>1 year), the year of transplant, which was categorized into three mutually exclusive groups (2017-2018, 2019-2020, and 2021-2022), HLA compatibility (allogeneic/haploidentical), blood group compatibility (compatible/not compatible), calculated CD34 dose, conditioning chemotherapy (myeloablative/intermediate/non-myeloablative), and 2) complications after the transplant and during follow-up: sepsis (yes/no) defined by clinical suspicion of infection (usually febrile neutropenia) and/or laboratory evidence of bacteremia (usually positive blood culture) and elevated inflammatory markers (such as fever and/or C-reactive protein and/or procalcitonin) and organ failure measured with the Sequential Organ Failure Assessment (SOFA) scale ^18^, CMV viral load (not detected/30 to 1000 copies/>1000 copies), acute GVHD (yes/no) which was considered if the patient presented cutaneous, intestinal and/or hepatic clinical signs of GVHD before day 100 of follow-up, and disease relapse (yes/no) (supplementary material).

### Statistical analysis

The analysis was performed in the statistical program Stata version 17. The variables were described in frequencies, percentages, measures of central tendency, and dispersion. The normality of quantitative variables was analyzed using the Shapiro-Wilk test. Hypothesis tests such as chi-square or Fisher’s exact test (as appropriate) were used when comparing mortality with categorical variables, and the Mann-Whitney U test when comparing it with numerical variables because they had an asymmetric distribution; a p-value <0.05 was considered significant.

To answer the research question, we used Cox proportional models. Because the analysis was exploratory, we first performed a crude analysis between each exposure variable and mortality at two years of follow-up; those variables with a p-value <0.2 were entered into the multivariate model where we calculated the adjusted hazard ratios (aHR) and their respective 95% confidence intervals (95% CI). In the second Cox model, we followed the same sequence, but the outcome variable was transplant-related mortality up to 100 days of follow-up. Collinearity was analyzed using the variance inflation factor (VIF) for each model; we considered a cutoff of 10 to identify potentially problematic collinearity. Both regression models showed VIF values below this threshold. We also evaluated the proportionality assumptions using Schoenfeld residuals. The sepsis variable was not included in the multivariate model up to 2 years of follow-up as it did not meet the proportionality assumptions (p = 0.037); this is because sepsis during the early period of HSCT generates a high risk of mortality, but this risk decreases as time passes since the patient’s immunity is restored over the months. Finally, the variables that were significant in the multivariate model up to two years of follow-up were presented in Kaplan-Meier survival graphs, and the differences between the survival functions were evaluated with the log-rank test.

### Ethical considerations

The research ethics guidelines according to the Declaration of Helsinki were followed. The protocol was approved by the HNERM ethics committee (verification code: 475-GRPR-ESSALUD-2024). The security and confidentiality of the clinical data obtained for this research were guaranteed. Informed consent was not required due to the observational and retrospective nature of the study, which only included the analysis of clinical records.

## RESULTS

### Population characteristics

Between January 2017 and May 2022, 744 hematopoietic stem cell transplants (HSCT) were performed at HNERM. Of these, 394 were allogeneic transplants (53% of the total) and the rest were autologous. 52 patients were excluded for the following criteria: (a) lack of a minimum 2-year follow-up (n=47), (b) incomplete clinical data in the records (n=5). The final cohort included 342 recipients of allogeneic HSCT.

The baseline characteristics showed a median age of 23 years (interquartile range [IQR]: 10-39), with 53% being female. The distribution by donor type was 52.9% compatible related and 47.1% haploidentical. The main indications were acute lymphoblastic leukemia (54.1%), acute myeloid leukemia (18.4%), and severe aplastic anemia (11.1%). Regarding patients with lymphoma, seven cases had a diagnosis of disease relapse after a previous autologous transplant. The rest were T/NK-cell non-Hodgkin lymphomas refractory to chemotherapy. The median time from diagnosis to transplant was 209 days (IQR: 299 - 519) (table 1).

**Table 1:** Characteristics of the study population

Characteristics		n (%)
Age^a^		23 (10‒39)
	≤11 years	93 (27.3)
	12 to 17 years	56 (16.4)
	18 to 49 years	160 (46.9)
	≥50 years	32 (9.4)
Sex		
	Female	183 (53.5)
	Male	159 (46.5)
Diagnosis		
	Acute lymphoblastic leukemia	185 (54.1)
	Acute myeloid leukemia	63 (18.4)
	Severe aplastic anemia	38 (11.2)
	Myelodysplastic syndrome	10 (2.9)
	Hodgkin and non-Hodgkin lymphoma	9 (2.6)
	Others	37 (10.8)
Time to transplant^a^		209 (299-519)
	≤1 year	209 (62.0)
	>1 year	128 (38.0)
Year of transplant^a^		
	2017 - 2018	133 (39.2)
	2019 - 2020	114 (33.6)
	2021 - 2022	92 (27.2)
Human leukocyte antigen compatibility		
	Compatible related (8/8-10 antigens)	181 (52.9)
	Haploidentical (4/8 antigens)	161 (47.1)
Dose of CD34+ cells administered (CD34+/kg)^a^		5.75 (7‒8)
ABO group incompatibility		
	No	286 (83.6)
	Yes	56 (16.4)
Type of conditioning chemotherapy		
	Myeloablative	239 (70.1)
	Intermediate	50 (14.7)
	Non-myeloablative	52 (15.2)
Days to transplant engraftment^a^		15 (13‒19)
Sepsis		
	No	300 (87.7)
	Yes	42 (12.3)
CMV viral load		
	Not detected	153 (50.3)
	30 to 1000 copies	95 (31.3)
	> 1000 copies	56 (18.4)
Graft-versus-host disease		
	No	184 (65.7)
	Yes	96 (34.3)
Disease relapse		
	No	235 (68.7)
	Yes	107 (31.3)
Other complications		
	No	243 (71.3)
	Yes	98 (28.7)
Time to death in the first hundred days^a^		25 (16‒42)
Death within one hundred days of follow-up		
	No	314 (91.8)
	Yes	28 (8.2)
Time to death at two years^a^		217 (81.5‒395.5)
Death within two years of follow-up		
	No	238 (69.6)
	Yes	104 (30.4)

a Median and interquartile range, ^b^ Categories are mutually exclusive. n: number of patients with information available for the evaluated variable, CMV: cytomegalovirus.

### Bivariate analysis regarding mortality

Of the total, 104 (30.4%) patients died post-HSCT, of which 28 (8.2%) occurred within the first 100 days and the remaining 76 died up to the second year post-HSCT. Death was more frequent in patients diagnosed with acute myeloid leukemia (42.9%, p=0.005), in those receiving a haploidentical transplant (38.5%, p=0.002), and in those who developed complications such as sepsis (76.2%, p=0.001) and disease relapse (62.6%, p=0.001) (table 2). Death at 100 days of follow-up was more frequent in those who received HSCT from a haploidentical donor (12.4%, p=0.007) and when there was no graft engraftment (76.9%, p<0.001) (supplementary material).

**Table 2:** Bivariate analysis between surviving and non-surviving patients

Characteristics		Survived n (%)	Did not survive n (%)	p-value
Age^a^		21 (10‒39)	27 (11‒45)	0.258^b^
Age groups				0.793^c^
	≤11 years	67 (72.0)	26 (28.0)	
	12 to 17 years	39 (69.6)	17 (30.4)	
	18 to 49 years	112 (70.0)	48 (30.0)	
	≥50 years	20 (62.5)	12 (37.5)	
Sex				0.134^c^
	Female	121 (66.1)	62 (33.9)	
	Male	117 (73.6)	42 (26.4)	
Diagnosis				0.005^d^
	Acute lymphoblastic leukemia	126 (68.1)	59 (31.9)	
	Acute myeloid leukemia	36 (57.1)	27 (42.9)	
	Severe aplastic anemia	35 (92.1)	3 (7.9)	
	Myelodysplastic syndrome	7 (70.0)	3 (30.0)	
	Hodgkin and non-Hodgkin lymphoma	6 (66.7)	3 (33.3)	
	Others	28 (75.7)	9 (24.3)	
Time to transplant^a^		292 (206‒458)	319 (224‒627.5)	0.249^b^
Time to transplant				0.544^c^
	≤1 year	147 (70.3)	62 (29.7)	
	>1 year	86 (67.2)	42 (32.8)	
Year of transplant				0.131^c^
	2017 - 2018	86 (64.7)	47 (35.3)	
	2019 - 2020	78 (68.4)	36 (31.6)	
	2021 - 2022	71 (77.2)	21 (22.8)	
Human leukocyte antigen compatibility				0.002^c^
	Compatible related (8/8-10 antigens)	139 (76.8)	42 (23.2)	
	Haploidentical (4/8 antigens)	99 (61.5)	62 (38.5)	
	Dose of CD34+ cells administered (CD34+/kg)^a^	7 (5.78‒8)	7 (5.31‒8)	0.912^b^
ABO group incompatibility				0.531^c^
	No	201 (70.3)	85 (29.7)	
	Yes	37 (66.1)	19 (33.9)	
Type of conditioning chemotherapy				0.072^c^
	Myeloablative	166 (69.5)	73 (30.5)	
	Intermediate	30 (60.0)	20 (40.0)	
	Non-myeloablative	42 (80.8)	10 (19.2)	
	Day to transplant engraftment^a^	15 (13‒19)	16 (13‒21)	0.120^b^
Sepsis				<0.001^c^
	No	228 (76.0)	72 (24.0)	
	Yes	10 (23.8)	32 (76.2)	
CMV viral load				0.646^c^
	Not detected	105 (68.6)	48 (31.4)	
	30 to 1000 copies	70 (73.7)	25 (26.3)	
	> 1000 copies	38 (67.9)	18 (32.1)	
Graft-versus-host disease				0.004^c^
	No	147 (79.9)	37 (20.1)	
	Yes	77 (80.2)	19 (19.8)	
Disease relapse				<0.001^c^
	No	198 (84.3)	37 (15.7)	
	Yes	40 (37.4)	67 (62.6)	
Other complications				0.773^c^
	No	170 (70.0)	73 (30.0)	
	Yes	67 (68.4)	31 (31.6)	

a Median and interquartile range, ^b^ Mann-Whitney U test, ^c^ Chi-square test, ^d^ Fisher’s exact test.

### Risk factors associated with post-HSCT death

In the multivariate Cox model, up to two years of follow-up, the risk factors associated with death were the haploidentical transplant source (aHR: 1.81; 95% CI: 1.21-2.72) compared to compatible related donors, and disease relapse (aHR: 4.17; 95% CI: 2.75-6.31) (table 3).

**Table 3:** Factors associated with all-cause mortality up to two years of follow-up

Variable		Crude model		Adjusted model	
		cHR (95% CI)	p-value	aHR (95% CI)	p-value
Age					
	≤11 years	Reference		-	
	12 to 17 years	1.21 (0.66‒2.23)	0.543	-	
	18 to 49 years	1.11 (0.69‒1.79)	0.673	-	
	≥50 years	1.52 (0.77‒3.01)	0.23	-	
Sex					
	Female	Reference		Reference	
	Male	0.77 (0.52‒1.14)	0.19	0.78 (0.53-1.17)	0.232
Type of conditioning chemotherapy					
	Myeloablative	Reference		-	
	Intermediate	1.38 (0.84‒2.26)	0.204	-	
	Non-myeloablative	0.61 (0.32‒1.19)	0.149	-	
Diagnosis					
	Acute lymphoblastic leukemia	Reference		Reference	
	Acute myeloid leukemia	1.47 (0.93‒2.32)	0.097	1.72 (1.08 - 2.74)	0.023
	Others	0.57 (0.34‒0.97)	0.037	0.90 (0.52-1.55)	0.712
Time to transplant					
	≤1 year	Reference		-	
	>1 year	1.12 (0.76‒1.65)	0.577	-	
Year of transplant					
	2017 - 2018	Reference		-	
	2019 - 2020	0.86 (0.56‒1.33)	0.495	-	
	2021 - 2022	0.62 (0.37‒1.04)	0.071	-	
Human leukocyte antigen (HLA) compatibility					
	Compatible related (8/8-10 antigens)	Reference		Reference	
	Haploidentical (4/8 antigens)	1.91 (1.29‒2.83)	0.001	1.81 (1.21-2.72)	0.004
ABO group incompatibility					
	No	Reference		-	
	Yes	1.10 (0.42‒2.89)	0.848	-	
Sepsis					
	No	Reference		Reference	
	Yes	5.48 (3.60‒8.33)	<0.001	-	
CMV viral load					
	Not detected	Reference		-	
	30 to 1000 copies	0.39 (0.13‒1.16)	0.089	-	
	> 1000 copies	0.33 (0.08‒1.44)	0.14	-	
Graft-versus-host disease					
	No	Reference		-	
	Yes	0.98 (0.57‒1.71)	0.955	-	
Disease relapse					
	No	Reference		Reference	
	Yes	4.68 (3.12‒7.02)	<0.001	4.17 (2.75-6.31)	<0.001
Other complications					
	No	Reference		-	
	Yes	1.10 (0.72‒1.67)	0.667	-	

Variables with a p-value <0.2 in the crude model were entered into the multivariate model.

cHR: crude hazard ratio, aHR: adjusted hazard ratio, HLA: human leukocyte antigen.

The Schoenfeld proportionality test had a p-value of 0.109. The collinearity analysis with VIF for the model was 1.09.

**Figure 1:**
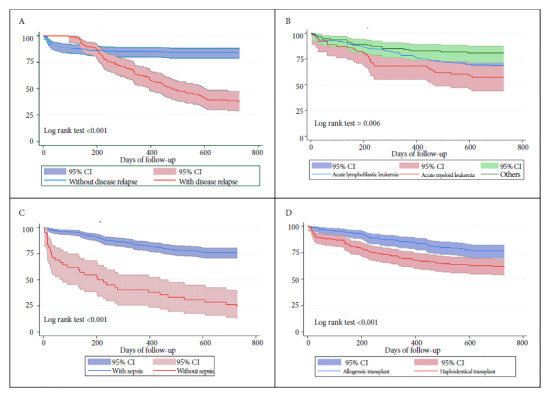
Kaplan-Meier survival curves according to the disease relapse category (A), the disease diagnosis (B), sepsis as a complication (C), and the type of transplant performed (D).

Figure 1 represents the survival function using Kaplan-Meier curves for the variables that were associated in the adjusted regression analysis.

Regarding transplant-related mortality at 100 days, in the multivariate Cox model, the associated risk factors were patients older than 50 years (aHR: 6.97; 95% CI: 1.18‒41.23) compared to patients 11 years or younger, those who received HSCT from a haploidentical donor (aHR: 3.57; 95% CI: 1.13‒11.24) compared to transplant from a compatible related donor, and those who had sepsis as a complication (aHR: 68.78; 95% CI: 19.32‒244.84) (table 4).

**Table 4:** Factors associated with transplant-related mortality up to 100 days of follow-up

Variable		Crude model		Adjusted model	
		cHR (95% CI)	p-value	aHR (95% CI)	p-value
Age					
	≤11 years	Reference		Reference	
	12 to 17 years	5.43 (1.47‒20.06)	0.011	3.02 (0.61‒15.06)	0.177
	18 to 49 years	2.16 (0.60‒7.74)	0.237	1.99 (0.48‒8.24)	0.343
	≥50 years	5.14 (1.23‒21.52)	0.025	6.97 (1.18‒41.23)	0.032
Gender					
	Female	Reference		-	
	Male	1.00 (0.47‒2.09)	0.99	-	
Type of conditioning chemotherapy					
	Myeloablative	Reference		-	
	Intermediate	1.13 (0.38‒3.35)	0.828	-	
	Non-myeloablative	1.97 (0.82‒4.76)	0.13	-	
Diagnosis					
	Acute lymphoblastic leukemia	Reference		-	
	Acute myeloid leukemia	1.70 (0.71‒4.05)	0.232	-	
	Others	0.83 (0.32‒2.17)	0.708	-	
Time to transplant					
	≤1 year	Reference		-	
	>1 year	1.24 (0.58‒2.61)	0.58	-	
Year of transplant					
	2017 - 2018	Reference		-	
	2019 - 2020	1.19 (0.49‒2.85)	0.702	-	
	2021 - 2022	1.16 (0.46 to 2.95)	0.751	-	
Human leukocyte antigen compatibility					
	Compatible related (8/8-10 antigens)	Reference		Reference	
	Haploidentical (4/8 antigens)	2.97 (1.31‒6.73)	0.009	3.57 (1.13‒11.24)	0.03
ABO group incompatibility					
	No	Reference		-	
	Yes	1.10 (0.42‒2.89)	0.848	-	
Sepsis					
	No	Reference		Reference	
	Yes	62.09 (17.60‒218.96)	<0.001	68.78 (19.32‒244.84)	<0.001
Graft-versus-host disease					
	No	Reference		-	
	Yes	1.05 (0.35‒3.12)	0.937	-	

Variables with a p-value <0.2 in the crude model were entered into the multivariate model.

cHR: crude hazard ratio; aHR: adjusted hazard ratio; HLA: Human Leukocyte Antigen; Ref: Reference value; CMV: cytomegalovirus;

The Schoenfeld proportionality test had a p-value of 0.301. The collinearity analysis with VIF for the model was 1.28.

## DISCUSSION

This is the first study in Peru to report outcomes after HSCT. Data from 342 allogeneic HSCT recipients between 2017 and 2022 were analyzed, and the following was identified: 1) death up to two years of follow-up occurred in 30.4% of patients; 2) the main risk factors associated with mortality were age over 50 years, diagnosis of acute myeloid leukemia, and complications such as sepsis and disease relapse; 3) transplant-related mortality (in the first 100 days post-HSCT) occurred in 8.2% of patients.

Mortality up to two years of follow-up was present in 30.4% of patients; these data are different from those reported in a multicenter study in 51 countries, which included 114,491 patients, where they report that death occurred in 45.8% of all patients ^19^. Another study by the European Group for Blood and Marrow Transplantation, which included data from 28,236 HSCT recipients, reported that mortality at two years of follow-up was 38.2% ^20^. Although in comparison with other studies, the mortality we report in our study appears to be lower, this may be due to multiple factors that must be considered, such as high heterogeneity in the population, the type of chemotherapy received, the follow-up time, and the use of unrelated donors, which limits the comparison of results with other studies and shows the clear need for more local information.

Sepsis was the main cause of death at 100 days post-HSCT follow-up. While the risk of infectious complications is a consequence of the prolonged immunosuppression produced by the antitumor radio-chemotherapy applied in the conditioning prior to HSCT ^1^, death related to infectious complications has decreased over the years ^21^. A study that analyzed data from 114,491 post-HSCT patients between 1980 and 2015 showed that mortality from bacterial and fungal infections has decreased over the years, although it also reports that the frequency of death due to infections of viral or unknown etiology did not change over time ^19^. The reduction in mortality from infectious complications may be due to a series of strategies that have been implemented over time, such as antibiotic, antifungal, and antiviral prophylaxis ^19^ and diagnostic methods by polymerase chain reaction ^21^. Although our study did not evaluate the specific impact of these interventions, their mention is relevant to contextualize global efforts to reduce infectious complications.

Despite the advances made in transplantation and the lower overall mortality related to complications, disease relapse, both early (occurring between 6 and 12 months) and late, remains a frequent complication post-HSCT ^22^. The most frequent types of cancer associated with post-HSCT relapse are acute myeloid leukemia, myelodysplastic syndrome, followed by lymphoblastic leukemias and lymphomas ^23^. This is likely the reason why, in our study, the group of patients diagnosed with acute myeloid leukemia had a higher risk of death at two years of follow-up. The loss of the graft-versus-tumor effect, due to immune evasion, has been proposed as a predisposing factor for this complication; other factors are: low-intensity conditioning regimens, advanced age of patients, and prolonged immunosuppression ^24^. Because disease recurrence is associated with higher mortality, it is essential to know which factors (especially modifiable ones) are associated with the development of recurrence in our population.

### Future research

Although Europe and North America are the regions of the world with the highest number of transplants per year, the number of procedures has increased in Latin America ^2^^,^^3^. In the United States, in 2022, approximately 8,500 transplants were performed, of which more than 3,500 were from unrelated donors ^5^. An unrelated donor is a person who, not being a direct relative, has HLA compatibility with the recipient. In Peru, unrelated donor HSCT is not yet performed. Initiating this type of transplant could increase the number of patients benefiting from the procedure, which would impact their survival.

Due to the need for more studies in Peru, we consider that these could focus on: 1) knowing the quality of life and non-hematological complications of patients with survival greater than two years; 2) evaluating which factors are associated with the waiting time between the indication for HSCT and the performance of the procedure; 3) identifying modifiable factors associated with the development of complications such as sepsis and disease recurrence; 4) replicating this first study prospectively and multicentrically to have a clearer idea of the characteristics of HSCT in Peru and 5) conducting research that seeks to know other factors associated with mortality at 5 and 10 years post-HSCT.

### Public health implications

Our findings reveal substantial challenges, but also key opportunities for the Peruvian health system. Among these, the need to strengthen infection prevention strategies stands out. The high incidence of infectious complications and the high mortality associated with sepsis underscore the urgency of implementing robust epidemiological surveillance protocols, especially focused on healthcare-associated infections and the early detection of multidrug-resistant pathogens. In this context, clinical follow-up by infectious disease specialists could play a crucial role in reducing these complications and improving clinical outcomes.

The high mortality in haploidentical recipients reinforces the need to implement a national unrelated donor program, like the Brazilian model that increased survival by 22% ^3^, as well as the need to investigate further why death occurs in this group of patients. Currently, Peru does not perform this type of transplant, which limits the options for patients without compatible family donors. According to a recent study ^25^, the average rate of HSCT in Latin America is 103 per 10 million inhabitants (range: 4-362); in Peru, the rate is 72, still below the regional average. This gap could be attributed to limited infrastructure: there are only ten accredited HSCT centers in Peru, seven of which are concentrated in the Peruvian capital and only four perform allogeneic transplants ^11^^,^^26^^,^^27^. The scarce availability of centers and their marked centralization represent a significant barrier to equitable access to this treatment.

This study has some limitations that should be considered when interpreting the results. First, due to the observational nature of our research, we could not determine causality between the association of variables. Second, although most patients who undergo HSCT do not usually have decompensated comorbidities, this data was not included; its absence in the analyze could prevent adjustments for baseline frailty and overestimate the effect of other variables. Third, since the reported results are from a single hospital center, they might not be generalizable to the entire Peruvian population, but it is worth mentioning that HNERM is one of the four centers that perform allogeneic HSCT in Peru and is the one that has performed the most transplants. Fourth, since we conducted a retrospective study, we could not analyze other outcomes of interest, such as quality of life and the development of chronic diseases after transplantation.

In conclusion, this study shows that post-HSCT mortality in Peru (8.2% at 100 days and 30.4% at 2 years) is comparable to international standards, with sepsis, haploidentical transplant, disease relapse, and age >50 years being the main associated factors. The results justify the implementation of standardized protocols for the prevention and management of infections by multidrug-resistant germs, unrelated donor programs, and an increase in the number of centers performing transplants. The sustainability of these advances will require investment in infrastructure, specialized training, and long-term follow-up systems.

## References

[1] Granot N, Storb R (2020). History of hematopoietic cell transplantation Challenges and progress. Haematologica.

[2] Malard F, Holler E, Sandmaier BM, Huang H, Mohty M (2023). Acute graft-versus-host disease. Nat Rev Dis Primer.

[3] Dambros VL, Gasparetto C, Costella G, Azevedo V, Trevizan S, Heck LH (2021). Análise dos transplantes de medula óssea realizados no brasil entre 2015 e 2020. Hematol Transfus Cell Ther.

[4] Saad A, de Lima M, Anand S, Bhatt VR, Bookout R, Chen G (2020). Hematopoietic cell transplantation, version 2 2020, NCCN Clinical practice guidelines in oncology. J Natl Compr Canc Netw.

[5] Cusatis R, Litovich C, Feng Z, Allbee-Johnson M, Shaw BE (2024). Current uses and outcomes of cellular therapies: CIBMTR Summary Slides, 2023 Summary Slides & Reports.

[6] Teira P, Battiwalla M, Ramanathan M, Barrett AJ, Ahn KW, Chen M (2016). Early cytomegalovirus reactivation remains associated with increased transplant-related mortality in the current era a CIBMTR analysis. Blood.

[7] Huang QS, Han TX, Fu HX, Meng H, Zhao P, Wu YJ (2024). Prognostic factors and outcomes in patients with septic shock after allogeneic hematopoietic stem cell transplantation. Transplant Cell Ther.

[8] De La Camara R (2016). CMV In hematopoietic stem cell transplantation. Mediterr J Hematol Infect Dis.

[9] Moreno M, Palacios CE, Cruz YC (2016). Primer trasplante haploidentico en Perú. Rev Fac Med Humana.

[10] von Elm E, Altman DG, Egger M, Pocock SJ, Gøtzsche PC, Vandenbroucke JP (2008). Declaración de la iniciativa STROBE (strengthening the reporting of observational studies in epidemiology) directrices para la comunicación de estudios observacionales. Rev Esp Salud Pública.

[11] Aranda-Gomero L, Pichardo-Rodriguez R, Fernandez-Vertíz I, Wong-Chang A (2022). Trasplante de células madre hematopoyéticas en el Perú: experiencia y desafíos del centro de trasplante más grande del Perú. Rev Fac Med Humana.

[12] EsSalud EEUU califica a Hospital Rebagliati de EsSalud como Primer Centro Trasplantador de alcance mundial.

[13] Agencia Peruana de Noticias Andina (2021). EsSalud realizó cerca de 100 trasplantes de médula ósea en lo que va del año.

[14] Agencia Peruana de Noticias Andina (2021). Hospital Rebagliati a la vanguardia en trasplante de médula ósea en Latinoamérica.

[15] Kong SG, Jeong S, Lee S, Jeong JY, Kim DJ, Lee HS (2021). Early transplantation-related mortality after allogeneic hematopoietic cell transplantation in patients with acute leukemia. BMC Cancer.

[16] Ministerio de Salud del Perú (2025). Sistema Informático Nacional de Defunciones (SINADEF).

[17] Registro Nacional de Identificación y Estado Civil (2025). Plataforma del Estado Peruano.

[18] Moreno R, Rhodes A, Piquilloud L, Hernandez G, Takala J, Gershengorn HB (2023). The sequential organ failure assessment (SOFA) Score has the time come for an update?. Crit Care.

[19] Styczynski J, Tridello G, Koster L, Iacobelli S, Van Biezen A (2020). Death after hematopoietic stem cell transplantation changes over calendar year time, infections and associated factors. Bone Marrow Transplant.

[20] Shouval R, Labopin M, Bondi O, Mishan-Shamay H, Shimoni A, Ciceri F (2015). Prediction of allogeneic hematopoietic stem-cell transplantation mortality 100 days after transplantation using a machine learning algorithm. J Clin Oncol.

[21] Ullmann AJ, Schmidt-Hieber M, Bertz H, Heinz WJ, Kiehl M, Krüger W (2016). Infectious diseases in allogeneic haematopoietic stem cell transplantation prevention and prophylaxis strategy guidelines 2016. Ann Hematol.

[22] Horowitz M, Schreiber H, Elder A, Heidenreich O, Vormoor J, Toffalori C (2018). Epidemiology and biology of relapse after stem cell transplantation. Bone Marrow Transplant.

[23] Ciurea SO, Kothari A, Sana S, Al Malki MM (2023). The mythological chimera and new era of relapse prediction post-transplant. Blood Rev.

[24] Gooley TA, Chien JW, Pergam SA, Hingorani S, Sorror ML, Boeckh M (2010). Reduced mortality after allogeneic hematopoietic-cell transplantation. N Engl J Med.

[25] Galeano S, Bonfim C, Karduss A, Jaimovich G, Gómez-De León A, Bettarello G (2025). Results of the latin american bone marrow transplantation society (LABMT) activity survey 2019-2022: the impact of the COVID-19 pandemic and the increase in related haploidentical donors. Bone Marrow Transplant.

[26] MINSA Dirección general de donaciones, trasplantes y banco de sangre (DIGDOT).

[27] Radio Nacional Hospital Nacional Dos de Mayo realizó con éxito primer trasplante de médula ósea.

