# Supplementary material for: Mortality and associated factors in post-stem cell transplant patients: a two-year cohort in a public hospital in Peru
Source: Rev Peru Med Exp Salud Publica. 2025 Sep 2;42(3):252–62. doi: 10.17843/rpmesp.2025.423.14389 (PMC12679968; doi:10.17843/rpmesp.2025.423.14389)
Supplement: Supplementary material. — Available in the electronic version of the RPMESP. [file rpmesp-42-03-14389-s001.docx]

**Tabla suplementaria 1:** Definición operacional de las variables.

| **Variables** | **Deficnión conceptual** | **Definición operacional** | **Escala de medición** | **Método de medición** | **Fuente de datos** |
| --- | --- | --- | --- | --- | --- |
| Edad | Edad cronológica al momento del TPH | Años cumplidos estratificados en: (1) ≤11 años, (2) 12 a 17 años, (3) 18 a 49 años, (4) ≥50 años | Ordinal | Registro en historia clínica (HC). | Historia clínica electrónica |
| Sexo | Sexo biologico | (1) Masculino; (2) Femenino | Nominal | Registro en historia clínica (HC). | Historia clínica electrónica |
| Diagnóstico | Diagnóstico oncohematológico que indica el procedimiento | (1) LLA-B; (2) LMA; (3) Anemia Aplásica; (4) SMD; (5) Linfomas (Hodgkin/No Hodgkin); (6) Otros (LMC, inmunodeficiencia, etc.). | Nominal | Confirmación por patología/citometría de flujo. | Historia clínica electrónica + reporte laboratorio |
| Tiempo hasta el trasplante | Periodo entre el diagnóstico y el trasplante | (1) <1 año; (2) ≥1 año. Calculado en días desde diagnóstico hasta infusión de células. | Ordinal | Fechas de diagnóstico y TPH en HC. | Historia clínica electrónica |
| Compatibilidad Antígeno Leucocitario Humano (HLA) | Grado de coincidencia de antígenos HLA entre donante y receptor. | (1) Matched emparentado (8/8-10 antígenos); (2) Haploidéntico (4/8 antígenos) | Nominal | Tipificación HLA por PCR-SSO/PCR-SSP. | Historia clínica electrónica |
| Compatibilidad ABO | Compatibilidad de grupo sanguíneo (A, B, O). | (1) Compatible; (2) No compatible. | Nominal | Prueba de aglutinación. | Historia clínica electrónica |
| Dosis celular CD34+ | Cantidad de células madre hematopoyéticas infundidas. | Número de células CD34 +/kg de peso del receptor (unidades: 10⁶/kg). | Continua | Citometría de flujo post-leucoféresis. | Registro de la UTPH |
| Régimen de acondicionamiento | Terapia pre-TPH para erradicar células malignas y suprimir inmunidad. | (1) Mieloablativo; (2) Intensidad reducida; (3) No mieloablativo. | Nominal | Protocolo de quimioterapia/radioterapia en HC. | Historia clínica electrónica |
| Prendimiento | Recuperación de neutrófilos post-TPH. | (1) Sí (neutrófilos ≥500/μL por 3 días consecutivos); (2) No. | Nominal | Hemograma diario | Registro de la UTPH |
|  |  | Día de prendimiento: día post-TPH en que se cumple el criterio. | Ordinal | Hemograma diario |  |
| Enfermedad Injerto contra Huésped | Reacción inmunológica del injerto contra tejidos del receptor. | (1) Sí (cutáneo/hepático/gastrointestinal); (2) No. Confirmación clínica ± biopsia. | Nominal | Criterios de Glucksberg | Historia clínica electrónica |
| Recaída de enfermedad | Reaparición de enfermedad post-TPH. | (0) No cursa con recaida de la enfermedad post-TPH (1) Cursa con recaída de la enfermedad post-TPH | Ordinal | Citometría de flujo + biopsia de médula ósea positiva a células malignas. | Laboratorio de patología |
| Sepsis | Infección con disfunción orgánica. | (1) Sí (≥2 criterios SOFA + sospecha clínica/microbiológica); (2) No. | Nominal | Hemocultivos y/o marcadores inflamatorios (PCR/procalcitonina) y/o fiebre y/o falla orgánica | Historia clínica electrónica |
| Mortalidad temprana | Muerte por cualquier causa post-TPH hasta los 100 días de seguimiento. | (0) No (1) Sí | Ordinal | Registro en HC + SINADEF. | Registro en HC + SINADEF. |
| Mortalidad tardía | Muerte por cualquier causa post-TPH hasta los dos años de seguimiento. | (0) No (1) Sí | Ordinal | Registro en HC + SINADEF. | Registro en HC + SINADEF. |
| Reactivación Citomegalovirus | Replicación detectable de CMV post-TPH. | Carga viral ≥30 copias/mL por PCR-RT. | Discreta | PCR cuantitativa (COBAS AmpliPrep/COBAS TaqMan). | Laboratorio de biología Molecular |

**Tabla suplementaria 2:** Análisis bivariado entre pacientes que sobrevivientes y no sobrevivientes a los 100 días de seguimiento

| **Características** | **Sobrevivió (%)** | **No sobrevivió (%)** | **Valor p** |
| --- | --- | --- | --- |
| Edad* | 22 (10 - 39) | 28 (13,5 - 47) | 0,186 ^a^ |
| Edad |  |  | 0,016 ^b^ |
| ≤11 años | 90 (85,4) | 3 (7,6) |  |
| 12 a 17 años | 47 (51,4) | 9 (4,6) |  |
| 18 a 49 años | 149 (146,9) | 11 (13,1) |  |
| ≥50 años | 27 (29,4) | 5 (2,6) |  |
| Sexo |  |  | 0,994 ^c^ |
| Femenino | 168 (91,8) | 15 (8,2) |  |
| Masculino | 146 (91,8) | 13 (8,2) |  |
| Diagnosis |  |  | 0,410 ^b^ |
| Leucemia lifoblástica aguda | 171 (92,4) | 14 (7,6) |  |
| Leucemia mieloide aguda | 55 (87,3) | 8 (12,7) |  |
| Anemia aplásica severa | 36 (94,7) | 2 (5,3) |  |
| Síndrome mielodisplásico | 8 (80,0) | 2 (20,0) |  |
| Linfoma de hodgkin y no hodgkin | 9 (100,0) | 0 (0,0) |  |
| Otros | 35 (94,6) | 2 (5,4) |  |
| Tiempo hasta el trasplante* | 296 (210 - 483) | 318 (131,5 - 693) | 0,772 ^a^ |
| Tiempo hasta el trasplante |  |  | 0,579 ^c^ |
| ≤1 año | 193 (92,3) | 16 (7,7) |  |
| >1 año | 116 (90,6) | 12 (9,4) |  |
| Año en el que se realizó el trasplante |  |  | 0,924 ^c^ |
| 2017 a 2018 | 123 (92,5) | 10 (7,5) |  |
| 2019 a 2020 | 104 (91,2) | 10 (8,8) |  |
| 2021 a 2022 | 84 (91,3) | 8 (8,7) |  |
| Compatibilidad antígeno leucocitario humano |  |  | 0,007 ^c^ |
| Emparentado compatible (8/8-10 antígenos) | 173 (95,6) | 8 (4,4) |  |
| Haploidéntico (4/8 antígenos) | 141 (87,6) | 20 (12,4) |  |
| Dosis de células CD34+ administrada (CD34+/kg)* | 7,29 (5,8 - 8) | 5.11 (4,57 - 7) | 0,023 ^a^ |
| Incompatibilidad del grupo ABO |  |  | 0,792 ^b^ |
| No | 263 (92,0) | 23 (8,0) |  |
| Sí | 51 (91,1) | 5 (8,9) |  |
| Tipo de quimioterapia de acondicionamiento |  |  | 0,302 ^b^ |
| Mieloablativo | 222 (92,9) | 17 (7,1) |  |
| Intermedio | 46 (92,0) | 4 (8,0) |  |
| No mieloablativo | 45 (86,5) | 7 (13,5) |  |
| Prendimiento del trasplante |  |  | <0,001 ^b^ |
| No | 3 (23,1) | 10 (76,9) |  |
| Sí | 311 (94,5) | 18 (5,5) |  |
| Día hasta el prendimiento del trasplante* | 15 (13 - 19) | 14.5 (12,5 - 19) | 0,419 ^a^ |
| Sepsis |  |  | <0,001 ^b^ |
| No | 256 (98,8) | 3 (1,2) |  |
| Sí | 11 (45,8) | 13 (54,2) |  |
| Carga viral de CMV |  |  | 0,092 ^c^ |
| No detectada | 137 (89,5) | 16 (10,5) |  |
| 30 a 1000 copias | 91 (95,8) | 4 (4,2) |  |
| > 1000 copias | 54 (96,4) | 2 (3,6) |  |
| Enfermedad injerto contra huésped |  |  | 0,999 ^b^ |
| No | 175 (95,1) | 9 (4,9) |  |
| Sí | 91 (94,8) | 5 (5,2) |  |
| Otras complicaciones |  |  | 0,001 ^c^ |
| No | 231 (95,1) | 12 (4,9) |  |
| Sí | 82 (83,7) | 16 (16,3) |  |

*Mediana y rango intercuartílico; a: Prueba estadística U de Mann Whitney; b: Prueba estadística exacta de Fisher; c: Prueba estadística Chi cuadrado.
