# Supplementary material for: Mortality and associated factors in post-stem cell transplant patients: a two-year cohort in a public hospital in Peru
Source: Rev Peru Med Exp Salud Publica. 2025 Sep 2;42(3):252–62. doi: 10.17843/rpmesp.2025.423.14389 (PMC12679968; doi:10.17843/rpmesp.2025.423.14389)
Supplement: Translation

# Translation: Mortalidad y factores asociados en pacientes postrasplante de células madre: cohorte de dos años en un hospital público del Perú

**Authors:** Mario Agramonte-Vilca, Karol Moscol-Chavez, Lourdes Aranda-Gomero, Alfredo Wong-Chang, Stalin Vilcarromero, Cesar Copaja-Corzo

**Affiliations:** 1 Universidad San Antonio Abad del Cusco, Cusco, Perú.; 2 Servicio de Hematología Especializada; Unidad de Trasplante de Progenitores Hematopoyéticos; Hospital Nacional Edgardo Rebagliati Martins, EsSalud, Lima, Perú.; 3 Servicio de Infectología, Hospital Nacional Edgardo Rebagliati Martins, EsSalud, Lima, Perú.; 4 Unidad de Investigación para la Generación y Síntesis de Evidencias en Salud, Universidad San Ignacio de Loyola, Lima, Perú.

**Keywords:** Infecciones Oportunistas Relacionadas con el Trasplante, Trasplante de Células Madre Hematopoyéticas, Enfermedad Injerto Contra Huésped, Sepsis, Leucemia Mieloide Aguda, Mortalidad Hospitalaria

## Abstract

**Objetivo.:**

Determinar la mortalidad y factores asociados en pacientes receptores de trasplante alogénico de precursores hematopoyéticos atendidos en el Hospital Nacional Edgardo Rebagliati Martins, entre enero 2017 y diciembre 2022.

**Materiales y métodos.:**

Se realizó un estudio de cohorte retrospectivo en pacientes sometidos a trasplante alogénico de precursores hematopoyéticos (TPH) entre 2017 y 2022. Se evaluó la muerte a los cien días y a los dos años, y sus factores asociados. Se emplearon modelos de regresión proporcional de Cox.

**Resultados.:**

Se incluyó a 342 pacientes pos-TPH, con una mediana de edad 23 años (rango intercuartílico: 10 a 39) de los cuales 53,5% eran mujeres. El diagnóstico más frecuente para el trasplante fue leucemia linfoblástica aguda con 54,1%. La mortalidad en los primeros 100 días pos-TPH fue del 8,2%, y a los dos años fue del 30,4%. En la regresión multivariada a los cien días, los factores asociados a mayor riesgo de mortalidad fueron la edad mayor a 50 años (HRa: 6,97; IC 95%: 1,18−41,23), aquellos receptores de trasplante haploidéntico (HRa: 3,57; IC 95%: 1,13−11,24) y la sepsis como complicación (HRa: 68,78; IC 95%: 19,32−244,84). En el análisis a los dos años, la leucemia mieloide aguda (1,72; IC 95%: 1,08-2,74) el trasplante haploidéntico (HRa:1,81; IC95%: 1,21-2,72) y la recaída de la enfermedad (HRa: 4,17; IC95%: 2,75-6,31) se asociaron a mayor riesgo de muerte.

**Conclusiones.:**

La mortalidad hallada fue baja y similar a la de países con mayores ingresos; sin embargo, es necesario formular intervenciones que reduzcan la incidencia de factores modificables como la sepsis.

## INTRODUCCIÓN

El trasplante de precursores hematopoyéticos (TPH), que consiste en la infusión de células madre pluripotenciales a individuos previamente acondicionados con radioquimioterapia antitumoral, constituye una intervención terapéutica vital para diversas patologías hematológicas malignas, no malignas e incluso, para algunas inmunodeficiencias primarias ^1^. El TPH permite curar y/o prolongar la vida de los pacientes oncohematológicos y es un procedimiento cada vez más frecuente en diversas regiones del mundo ^1^^-^^3^. Esto se debe a la inclusión de pacientes con mayor edad, regímenes de acondicionamiento quimioterápico menos tóxico, un mayor número de donantes no emparentados, avances en la tipificación de los antígenos leucocitarios humanos (HLA) de alta resolución, y uso de nuevos fármacos inmunosupresores ^1^^,^^4^.

Sin embargo, el TPH también conlleva riesgos y no está exento de complicaciones ^1^. Entre ellos, el riesgo de infecciones debido a la inmunosupresión inducida (radioquimioterapia previo al trasplante), el riesgo de toxicidad secundaria a quimioterápicos, la enfermedad de injerto contra huésped (EICH), la falla del injerto y la recurrencia de la malignidad ^1^^,^^4^. Como dato importante, el centro para la investigación del trasplante de médula y sangre de Estados Unidos (CIBMTR por sus siglas en inglés), entre el 2016 al 2021, reportaron que en los primeros 100 días de seguimiento posteriores al TPH, las infecciones, el fallo orgánico y la recaída de enfermedad subyacente contribuyeron a la mortalidad en 25%, 24% y 23% respectivamente ^5^. Las infecciones de causa bacteriana son más frecuentes en el periodo temprano posterior al trasplante mientras que la reactivación de las formas virales latentes ocurre habitualmente en fases posteriores ^6^^,^^7^. Además, el EICH agudo de severidad moderada a grave, ejerce un efecto sinérgico en la reactivación del citomegalovirus (CMV) y se ha asociado a deterioro en la calidad de vida en los sobrevivientes ^2^^,^^8^.

En Perú el TPH se ha realizado desde 1994, si bien con el paso de los años el número de trasplantes se ha incrementado en gran medida ^9^ la investigación en América Latina y sobre todo en Perú ha sido limitada. La unidad detrasplante de progenitores hematopoyéticos (UTPH) del Hospital Nacional Edgardo Rebagliati Martins (HNERM) es la más grande en Perú por lo que es un centro de referencia nacional. Dada la relevancia del tema y la ausencia de información, analizar los datos de la UTPH del HNERM brindara un primer acercamiento sobre los trasplantes en Perú que podrá generar información para la toma de decisiones. En tal sentido, este estudio tiene como objetivo determinar la mortalidad y factores asociados en pacientes receptores de trasplante alogénico de precursores hematopoyéticos atendidos en el HNERM, entre enero 2017 y diciembre 2022.

**MENSAJE CLAVE**

Motivación para realizar el estudio. En el Perú se ha incrementado el número de pacientes tratados mediante trasplante de precursores hematopoyéticos (TPH) pero la información sobre los resultados posteriores al TPH es limitada o inexistente.

Principales hallazgos. Entre 2017 y 2022 en el Hospital Nacional Edgardo Rebagliati Martins se realizaron 744 TPH, de los cuales 394 fueron alogénicos. La mortalidad a los 100 días después del TPH fue de 8,2% y a los dos años, de 30,4%, estas cifras fueron bajas y comparables con las reportadas en países de mayores ingresos. Los principales factores asociados a la mortalidad fueron la sepsis y la recaída de la enfermedad.

Implicancias. Es necesaria una mayor inversión y la implementación de estrategias más eficaces en salud pública para seguir aumentando el número de trasplantes y mejorar la supervivencia a largo plazo.

## MATERIALES Y MÉTODOS

### Diseño y entorno del estudio

Se realizó un estudio de cohorte retrospectivo mediante la revisión de historias clínicas de pacientes atendidos en la UTPH del HNERM en Perú. El manuscrito fue redactado siguiendo las directrices de Strengthening the Reporting of Observational Studies in Epidemiology (STROBE) para informar estudios observacionales ^10^.

En Perú hay 10 centros de trasplante de progenitores hematopoyéticos acreditados por el Ministerio de Salud (MINSA). Siete de ellos se encuentran en la capital peruana y tres en provincias. El HNERM es uno de los cuatro centros acreditados para trasplante alogénico ^11^, realiza trasplantes para enfermedades oncohematológicas y otros diagnósticos no oncológicos, como aplasia medular o inmunodeficiencias primarias. La UTPH ha realizado desde 1994 más de 1600 trasplantes, entre autólogos y alogénicos (familiar idéntico o no idéntico, este último también llamado haploidéntico), y es una unidad especializada de referencia nacional ^12^^-^^14^.

### Población

Se incluyó a todos los pacientes receptores de trasplante alogénico, entre enero de 2017 y mayo del 2022, de todas las edades. Además, el seguimiento del paciente debía ser superior a 730 días (dos años) y contar con el registro del resultado final del seguimiento (sobrevive o no sobrevive). Se excluyeron historias clínicas de pacientes que no completaron dicho seguimiento al momento del estudio. Se analizaron los datos de todos los pacientes que cumplían con los criterios de inclusión.

### Procedimientos y técnicas

Luego de obtener la aprobación del comité de ética y la autorización de la gerencia clínica del HNERM se accedió al registro de pacientes de la UTPH. Este es un registro digital en Excel elaborado a partir del seguimiento médico de los pacientes (periodo pre y postrasplante) y realizado por médicos especialistas en hematología de la UTPH. La base de datos fue gestionada en Microsoft Excel. Para asegurar la integridad y la precisión de los datos, el investigador principal, descargó la base y realizó una doble verificación cotejando la información registrada en la base con las historias clínicas electrónicas. Se implementó una limpieza de datos sistemática para detectar y corregir errores, como valores atípicos (ej. una hemoglobina de 87 g/dL), los cuales fueron validados y ajustados según el registro original de la historia clínica electrónica. En cuanto a los datos faltantes, se realizó una búsqueda exhaustiva en las historias clínicas electrónicas; si un dato no era localizable, se dejó en blanco y se analizó como dato perdido, sin imputación. Finalmente, para garantizar la confidencialidad, se eliminó toda la información personal y se asignaron códigos de estudio aleatorios antes de proceder al análisis estadístico.

En el HNERM se realizan trasplantes de progenitores hematopoyeticos autólogos y alogénicos de donante emparentado: con compatibilidad HLA idéntica (coincidencia del 100% HLA) y haploidentico (50% de los HLA), además de la identificación de anticuerpos donante-específico, según corresponda. Todos los pacientes que reciben el TPH se someten a estudios como ecocardiograma, espirometría, desfocalización odontológica y otorrinolaringológica. Además de un estudio completo de serología para toxoplasma, herpesvirus humanos, sífilis, VIH, hepatitis B y C, análisis bioquímico y evaluación de acceso venoso. Los pacientes con enfermedad oncohematológica antes de ser sometidos a TPH, deben encontrarse en remisión completa de la enfermedad.

En cuanto al procedimiento de trasplante: el tratamiento de acondicionamiento, que es el esquema de quimioterapia con o sin radioterapia administrado antes de la infusión de los precursores hematopoyéticos, puede tener intención mieloablativa, no mieloablativa o ser de intensidad reducida según la combinación y dosis de antineoplásicos e inmunosupresores. Los esquemas habitualmente utilizados son, por ejemplo: ciclofosfamida 50 mg/kg/dia y TBI 1200 cGy (centigray) como mieloablativos y fludarabina 30 mg/m^2^/día, busulfan 0,8mg/kg c/6h (por 12 dosis) como de intensidad reducida o intermedio. En paralelo, los donantes reciben desde cuatro días antes de la infusión de *stem cells*, factor estimulante de colonias (FEC 10 mcg/kg) para posteriormente ser colectados. La fuente de precursores hematopoyéticos utilizada es sangre periférica y se colecta mediante leucoféresis. En cuanto a la profilaxis para el EICH, los receptores emparentados compatibles (8/8-10/10 antígenos HLA) reciben desde el día −1 pos-TPH, ciclosporina 5 mg/kg/día y luego en el día +1 pos-TPH, metotrexato 15 mg/m^2,^ luego, en los días +3, +6 y +11 pos-TPH, metotrexato 10mg/m^2^. A su vez, los receptores de trasplante haploidéntico (4/8 antígenos HLA) reciben en el día +3 y +4 pos- TPH, ciclofosfamida 50 mg/kg/día y tacrolimus 0,075 mg/kg, asociado a micofenolato 15 mg/kg/dosis a partir del día +5 pos-TPH. Para prevenir infecciones virales se administra aciclovir.

### Variables

*Variable de resultado*

La mortalidad tardía se consideró cuando la muerte ocurrió posterior al TPH hasta los 730 días del seguimiento (dos años). La mortalidad temprana fue considerada como la muerte dentro de los primeros 100 días de seguimiento tras el trasplante ^15^. Para el registro de la mortalidad se evaluaron las historias clínicas digitales de los pacientes de EsSalud (Seguro Social en Salud), la ficha electrónica del Sistema de Información de Defunciones (SINADEF) ^16^ y el Registro Nacional de Identificación y Estado Civil (RENIEC) del Perú ^17^. Si bien el acceso web a ambas fuentes es libre, la verificación de defunciones es de acceso restringido (sólo el personal autorizado puede acceder a la información). En nuestro caso, la solicitud de acceso se gestionó mediante trámites administrativos con EsSalud.

*Variables de exposición*

Las variables de exposición se agruparon en: 1) características clínicas: edad (≤11/12-17/18-49/≥50), sexo (masculino/femenino), diagnóstico hematológico (leucemia linfoblástica aguda/leucemia mieloide aguda/anemia aplásica severa/síndrome mielodisplásico/linfoma de Hodgkin y no Hodgkin/otros) debido al reducido número de observaciones para el análisis de regresión se decidió recategorizar esta variable (leucemia linfoblástica aguda/leucemia mieloide aguda/otros), tiempo desde el diagnostico hasta el trasplante (≤1 año/>1 año), el año del trasplante, que fue categorizada en tres grupos mutuamente excluyentes (2017-2018, 2019-2020 y 2021-2022), compatibilidad según HLA (alogénico/haploidéntico), compatibilidad de grupo sanguíneo (compatible/no compatible), dosis calculada de CD34, quimioterapia de acondicionamiento (mieloablativo/intermedio/no mieloablativo) y 2) complicaciones luego del trasplante y durante el seguimiento: sepsis (sí/no) definido por la sospecha clinica de infección (generalmente, neutropenia febril) y/o laboratorial de bacteriemia (generalmente, hemocultivo positivo) y marcadores inflamatorios elevados (como fiebre y/o proteína C reactiva y/o procalcitonina) y la falla organica medida con la escala de Sequential Organ Failure Assessment (SOFA) ^18^, carga viral de CMV (no detectada/30 a 1000 copias/>1000 copias), EICH agudo (sí/no) que fue considerado si el paciente presentó clínica cutánea, intestinal y/o hepática de EICH antes del día 100 de seguimiento y recaida de la enfermedad (sí/no) (material suplementario).

### Análisis estadístico

El análisis se realizó en el programa estadístico Stata versión 17. Las variables fueron descritas en frecuencias, porcentajes, medidas de tendencia central y de dispersión. Se analizó la normalidad de las variables cuantitativas mediante la prueba de Shapiro-Wilk. Se emplearon pruebas de hipótesis como chi-cuadrado o exacta de Fisher (según correspondía) al comparar la mortalidad con variables categóricas y U de Mann-Whitney al compararla con variables numéricas debido a que tuvieron una distribución asimétrica, se consideró un valor p<0,05 como significativo.

Para responder a la pregunta de investigación empleamos modelos proporcionales de Cox. Debido a que el análisis fue exploratorio, primero realizamos un análisis crudo entre cada variable de exposición y la mortalidad a los dos años de seguimiento, aquellas variables con un valor de p<0,2 ingresaron al modelo multivariado donde calculamos los *hazard ratio* ajustados (HRa) y sus respectivos intervalos de confianza del 95% (IC 95%). En el segundo modelo de Cox seguimos la misma secuencia, pero la variable de resultado fue la mortalidad relacionada al trasplante hasta los 100 días de seguimiento. Se analizó la colinealidad mediante el factor de inflación de la varianza (VIF) para cada modelo, consideramos un punto de corte de 10 para identificar una colinealidad potencialmente problemática. Ambos modelos de regresión mostraron valores de VIF inferiores a este umbral, además evaluamos los supuestos de proporcionalidad mediante residuos de Schoenfeld. La variable sepsis no fue incluida en el modelo multivariado hasta los 2 años de seguimiento ya que no cumplía con los supuestos de proporcionalidad (p = 0,037), esto se debe a que la sepsis durante el periodo temprano del TPH genera un alto riesgo de mortalidad, pero este riesgo baja conforme pasa el tiempo ya que la inmunidad del paciente se restablece con el paso de los meses. Finalmente, las variables que fueron significativas en el modelo multivariado hasta los dos años de seguimiento fueron presentadas en gráficos de supervivencia de Kaplan-Meier y las diferencias entre las funciones de supervivencia fueron evaluadas con la prueba de log-rank.

### Criterios éticos

Se siguieron las pautas de ética en investigación de acuerdo con la declaración de Helsinki. El protocolo fue aprobado por el comité de ética del HNERM (código de verificación: 475-GRPR-ESSALUD-2024). Se garantizó la seguridad y confidencialidad de los datos clínicos obtenidos para esta investigación. No se requirió consentimiento informado debido a la naturaleza observacional y retrospectiva del estudio que solo incluía el análisis de historias clínicas.

## RESULTADOS

### Características de la población

Entre enero de 2017 y mayo de 2022, se realizaron 744 trasplantes de progenitores hematopoyéticos (TPH) en el HNERM. De estos, 394 fueron trasplantes alogénicos (53% del total) y el resto fueron autólogos. Se excluyeron 52 pacientes por los siguientes criterios: (a) falta de seguimiento mínimo de 2 años (n=47), (b) datos clínicos incompletos en las historias (n=5). La cohorte final incluyó 342 pacientes receptores de TPH alogénico.

Las características basales mostraron una mediana de edad de 23 años (rango intercuartílico [RIC]: 10-39), con 53% de sexo femenino. La distribución por tipo de donante fue 52,9% emparentado compatible y 47,1% haploidéntico. Las indicaciones principales fueron leucemia linfoblástica aguda (54,1%), leucemia mieloide aguda (18,4%) y anemia aplásica severa (11,1%). Con respecto a los pacientes con linfoma, siete casos tenían diagnóstico de recaída de enfermedad posterior a trasplante autólogo previo. El resto eran linfomas no Hodgkin de células T/NK refractarios a quimioterapia. El tiempo mediano desde el diagnóstico hasta el trasplante fue de 209 días (RIC: 299 - 519) (tabla 1).

**Tabla 1:** Características de la población de estudio.

Características		n (%)
Edad^a^		23 (10‒39)
	≤11 años	93 (27,3)
	12 a 17 años	56 (16,4)
	18 a 49 años	160 (46,9)
	≥50 años	32 (9,4)
Sexo		
	Femenino	183 (53,5)
	Masculino	159 (46,5)
Diagnóstico		
	Leucemia linfoblástica aguda	185 (54,1)
	Leucemia mieloide aguda	63 (18,4)
	Anemia aplásica severa	38 (11,2)
	Síndrome mielodisplásico	10 (2,9)
	Linfoma de Hodgkin y no Hodgkin	9 (2,6)
	Otros	37 (10,8)
Tiempo hasta el trasplante^a^		209 (299-519)
	≤1 año	209 (62,0)
	>1 año	128 (38,0)
Año en el que se realizó el trasplante^b^		
	2017 - 2018	133 (39,2)
	2019 - 2020	114 (33,6)
	2021 - 2022	92 (27,2)
Compatibilidad antígeno leucocitario humano		
	Emparentado compatible (8/8-10 antígenos)	181 (52,9)
	Haploidéntico (4/8 antígenos)	161 (47,1)
Dosis de células CD34+ administrada (CD34+/kg)^a^		5,75 (7‒8)
Incompatibilidad del grupo ABO		
	No	286 (83,6)
	Sí	56 (16,4)
Tipo de quimioterapia de acondicionamiento		
	Mieloablativo	239 (70,1)
	Intermedio	50 (14,7)
	No mieloablativo	52 (15,2)
Días hasta el prendimiento del trasplante^a^		15 (13‒19)
Sepsis		
	No	300 (87,7)
	Sí	42 (12,3)
Carga viral de CMV		
	No detectada	153 (50,3)
	30 a 1000 copias	95 (31,3)
	> 1000 copias	56 (18,4)
Enfermedad injerto contra huésped		
	No	184 (65,7)
	Sí	96 (34,3)
Recaída de enfermedad		
	No	235 (68,7)
	Sí	107 (31,3)
Otras complicaciones		
	No	243 (71,3)
	Sí	98 (28,7)
Tiempo hasta la muerte en los primeros cien días^a^		25 (16‒42)
Muerte dentro de los cien días de seguimiento		
	No	314 (91,8)
	Sí	28 (8,2)
Tiempo hasta la muerte a los dos años^a^		217 (81,5‒395,5)
Muerte dentro de los dos años de seguimiento		
	No	238 (69,6)
	Sí	104 (30,4)

a Mediana y rango intercuartílico, ^b^ Las categorías son mutuamente excluyentes. n: número de pacientes que contaban con información acerca de la variable evaluada, CMV: citomegalovirus.

### Análisis bivariado con respecto a la mortalidad

Del total, 104 (30,4%) pacientes fallecieron pos-TPH, de los cuales 28 (8,2%) ocurrieron dentro de los primeros 100 días y los 76 restantes fallecieron hasta el segundo año pos-TPH. La muerte fue más frecuente en pacientes con diagnóstico de leucemia mieloide aguda (42,9%, p=0,005), en aquellos receptores de trasplante de tipo haploidéntico (38,5%, p=0,002) y en aquellos que desarrollaron complicaciones como sepsis (76,2%, p=0,001) y recaída de la enfermedad (62,6%, p=0,001) (tabla 2). La muerte a los 100 días de seguimiento fue más frecuente en aquellos que recibieron el TPH de un donante haploidéntico (12,4% p=0,007) y cuando no hubo prendimiento del injerto (76,9%, p<0,001) (material suplementario).

**Tabla 2:** Análisis bivariado entre pacientes sobrevivientes y no sobrevivientes.

Características		Sobrevivió n (%)	No sobrevivió n (%)	Valor de p
Edad^a^		21 (10‒39)	27 (11‒45)	0,258^b^
Grupos de edad				0,793^c^
	≤11 años	67 (72,0)	26 (28,0)	
	12 a 17 años	39 (69,6)	17 (30,4)	
	18 a 49 años	112 (70,0)	48 (30,0)	
	≥50 años	20 (62,5)	12 (37,5)	
Sexo				0,134^c^
	Femenino	121 (66,1)	62 (33,9)	
	Masculino	117 (73,6)	42 (26,4)	
Diagnóstico				0,005^d^
	Leucemia linfoblástica aguda	126 (68,1)	59 (31,9)	
	Leucemia mieloide aguda	36 (57,1)	27 (42,9)	
	Anemia aplásica severa	35 (92,1)	3 (7,9)	
	Síndrome mielodisplásico	7 (70,0)	3 (30,0)	
	Linfoma de Hodgkin y no Hodgkin	6 (66,7)	3 (33,3)	
	Otros	28 (75,7)	9 (24,3)	
Tiempo hasta el trasplante^a^		292 (206‒458)	319 (224‒627,5)	0,249^b^
Tiempo hasta el trasplante				0,544^c^
	≤1 año	147 (70,3)	62 (29,7)	
	>1 año	86 (67,2)	42 (32,8)	
Año en el que se realizó el trasplante				0,131^c^
	2017 - 2018	86 (64,7)	47 (35,3)	
	2019 - 2020	78 (68,4)	36 (31,6)	
	2021 - 2022	71 (77,2)	21 (22,8)	
Compatibilidad antígeno leucocitario humano				0,002^c^
	Emparentado compatible (8/8-10 antígenos)	139 (76,8)	42 (23,2)	
	Haploidéntico (4/8 antígenos)	99 (61,5)	62 (38,5)	
	Dosis de células CD34+ administrada (CD34+/kg)^a^	7 (5,78‒8)	7 (5,31‒8)	0,912^b^
Incompatibilidad del grupo ABO				0,531^c^
	No	201 (70,3)	85 (29,7)	
	Sí	37 (66,1)	19 (33,9)	
Tipo de quimioterapia de acondicionamiento				0,072^c^
	Mieloablativo	166 (69,5)	73 (30,5)	
	Intermedio	30 (60,0)	20 (40,0)	
	No mieloablativo	42 (80,8)	10 (19,2)	
Día hasta el prendimiento del trasplante		15 (13‒19)	16 (13‒21)	0,120^b^
Sepsis				<0,001^c^
	No	228 (76,0)	72 (24,0)	
	Sí	10 (23,8)	32 (76,2)	
Carga viral de CMV				0,646^c^
	No detectada	105 (68,6)	48 (31,4)	
	30 a 1000 copias	70 (73,7)	25 (26,3)	
	> 1000 copias	38 (67,9)	18 (32,1)	
Enfermedad injerto contra huésped				0,004^c^
	No	147 (79,9)	37 (20,1)	
	Sí	77 (80,2)	19 (19,8)	
Recaída de enfermedad				<0,001^c^
	No	198 (84,3)	37 (15,7)	
	Sí	40 (37,4)	67 (62,6)	
Otras complicaciones				0,773^c^
	No	170 (70,0)	73 (30,0)	
	Sí	67 (68,4)	31 (31,6)	

a Mediana y rango intercuartílico, ^b^ prueba U de Mann-Whitney, ^c^ prueba chi-cuadrado, ^d^ prueba exacta de Fisher.

### Factores de riesgo asociados a muerte pos-TPH

En el modelo multivariado de Cox, hasta los dos años de seguimiento, los factores de riesgo asociados a muerte fueron la fuente de trasplante haploidéntico (HRa: 1,81; IC 95%: 1,21-2,72) en comparación con los emparentados compatibles y la recaída de la enfermedad (HRa: 4,17; IC 95%: 2,75-6,31) (tabla 3).

**Tabla 3:** Factores asociados a mortalidad por todas las causas hasta dos años de seguimiento.

Variable		Modelo crudo		Modelo ajustado	
		HRc (IC 95%)	Valor de p	HRa (IC 95%)	Valor de p
Edad					
	≤11 años	Referencia		-	
	12 a 17 años	1,21 (0,66‒2,23)	0,543	-	
	18 a 49 años	1,11 (0,69‒1,79)	0,673	-	
	≥50 años	1,52 (0,77‒3,01)	0,230	-	
Sexo					
	Femenino	Referencia		Referencia	
	Masculino	0,77 (0,52‒1,14)	0,190	0,78 (0,53-1,17)	0,232
Tipo de quimioterapia de acondicionamiento					
	Mieloablativo	Referencia		-	
	Intermedio	1,38 (0,84‒2,26)	0,204	-	
	No mieloablativo	0,61 (0,32‒1,19)	0,149	-	
Diagnóstico					
	Leucemia linfoblástica aguda	Referencia		Referencia	
	Leucemia mieloide aguda	1,47 (0,93‒2,32)	0,097	1,72 (1,08 - 2,74)	0,023
	Otros	0,57 (0,34‒0,97)	0,037	0,90 (0,52-1,55)	0,712
Tiempo hasta el trasplante					
	≤1 año	Referencia		-	
	>1 año	1,12 (0,76‒1,65)	0,577	-	
Año en el que se realizó el trasplante					
	2017 - 2018	Referencia		-	
	2019 - 2020	0,86 (0,56‒1,33)	0,495	-	
	2021 - 2022	0,62 (0,37‒1,04)	0,071	-	
Compatibilidad antígeno leucocitario humano (HLA)					
	Emparentado compatible (8/8-10 antígenos)	Referencia		Referencia	
	Haploidéntico (4/8 antígenos)	1,91 (1,29‒2,83)	0,001	1,81 (1,21-2,72)	0,004
Incompatibilidad del grupo ABO					
	No	Referencia		-	
	Sí	1,10 (0,42‒2,89)	0,848	-	
Sepsis					
	No	Referencia		Referencia	
	Sí	5,48 (3,60‒8,33)	<0,001	-	
Carga viral de CMV					
	No detectada	Referencia		-	
	30 a 1000 copias	0,39 (0,13‒1,16)	0,089	-	
	> 1000 copias	0,33 (0,08‒1,44)	0,140	-	
Enfermedad injerto contra huésped					
	No	Referencia		-	
	Sí	0,98 (0,57‒1,71)	0,955	-	
Recaída de enfermedad					
	No	Referencia		Referencia	
	Sí	4,68 (3,12‒7,02)	<0,001	4,17 (2,75-6,31)	<0,001
Otras complicaciones					
	No	Referencia		-	
	Sí	1,10 (0,72‒1,67)	0,667	-	

Ingresaron al modelo multivariado aquellas variables con un valor de p<0,2 en el modelo crudo.

HRc: hazard ratio crudo, HRa: hazard ratio ajustado, HLA: antígeno leucocitario humano.

El test de proporcionalidad de Schoenfeld tuvo un valor de p de 0,109. El analisis de colinealidad con VIF del modelo fue de 1.09.

La figura 1 representa la función de supervivencia mediante las curvas de Kaplan-Meier para las variables que resultaron asociadas en el análisis de regresión ajustado.

**Figura 1:** Curvas de supervivencia de Kaplan-Meier según la categoría de recaída de la enfermedad (A), el diagnóstico de enfermedad (B), la sepsis como complicación (C) y el tipo de trasplante realizado (D).

Con respecto a la mortalidad relacionada con el trasplante a los 100 días, en el modelo multivariado de Cox, los factores de riesgo asociados fueron, aquellos pacientes mayores de 50 años (HRa: 6,97; IC 95%: 1,18‒41,23) en comparación con los pacientes de 11 años o menos, en aquellos que recibieron el TPH de un donante haploidéntico (HRa: 3,57; IC 95%: 1,13‒11,24) en comparación al trasplante de un donante emparentado compatible y quienes tuvieron sepsis como complicación (HRa: 68,78; IC 95%: 19,32‒244,84) (tabla 4).

**Tabla 4:** Factores asociados a la mortalidad por causas relacionadas al trasplante hasta los 100 días de seguimiento.

Variable		Modelo crudo		Modelo ajustado	
		HRc (IC 95%)	Valor de p	HRa (IC 95%)	Valor de p
Edad					
	≤11 años	Referencia		Referencia	
	12 a 17 años	5,43 (1,47‒20,06)	0,011	3,02 (0,61‒15,06)	0,177
	18 a 49 años	2,16 (0,60‒7,74)	0,237	1,99 (0,48‒8,24)	0,343
	≥50 años	5,14 (1,23‒21,52)	0,025	6,97 (1,18‒41,23)	0,032
Género					
	Femenino	Referencia		-	
	Masculino	1,00 (0,47‒2,09)	0,990	-	
Tipo de quimioterapia de acondicionamiento					
	Mieloablativo	Referencia		-	
	Intermedio	1,13 (0,38‒3,35)	0,828	-	
	No mieloablativo	1,97 (0,82‒4,76)	0,130	-	
Diagnostico					
	Leucemia linfoblástica aguda	Referencia		-	
	Leucemia mieloide aguda	1,70 (0,71‒4,05)	0,232	-	
	Otros	0,83 (0,32‒2,17)	0,708	-	
Tiempo hasta el trasplante					
	≤1 año	Referencia		-	
	>1 año	1,24 (0,58‒2,61)	0,580	-	
Año en el que se realizó el trasplante					
	2017 - 2018	Referencia		-	
	2019 - 2020	1,19 (0,49‒2,85)	0,702	-	
	2021 - 2022	1,16 (0,46 to 2,95)	0,751	-	
Compatibilidad antígeno leucocitario humano					
	Emparentado compatible (8/8-10 antígenos)	Referencia		Referencia	
	Haploidéntico (4/8 antígenos)	2,97 (1,31‒6,73)	0,009	3,57 (1,13‒11,24)	0,030
Incompatibilidad del grupo ABO					
	No	Referencia		-	
	Sí	1,10 (0,42‒2,89)	0,848	-	
Sepsis					
	No	Referencia		Referencia	
	Sí	62,09 (17,60‒218,96)	<0,001	68,78 (19,32‒244,84)	<0,001
Enfermedad injerto contra huésped					
	No	Referencia		-	
	Sí	1,05 (0,35‒3,12)	0,937	-	

Ingresaron al modelo multivariado aquellas variables con un valor de p<0,2 en el modelo crudo.

HRc: hazard ratio crudo; HRa: hazard ratio ajustado; HLA: Antígeno Leucocitario Humano; Ref: Valor de referencia; CMV: citomegalovirus; El test de proporcionalidad de Schoenfeld tuvo un valor p de 0,301. El analisis de colinealidad con VIF del modelo fue de 1,28

## DISCUSIÓN

Este es el primer estudio en Perú que reporta desenlaces posteriores al TPH. Se analizó los datos de 342 pacientes receptores de TPH alogénico entre 2017 y 2022, y se identificó lo siguiente: 1) la muerte hasta los dos años de seguimiento ocurrió en el 30,4% de los pacientes; 2) los principales factores de riesgo asociados a mortalidad fueron la edad mayor a 50 años, el diagnóstico de leucemia mieloide aguda y complicaciones como la sepsis y la recaída de la enfermedad; 3) la mortalidad relacionada al trasplante (en los primeros 100 días pos-TPH) ocurrió en el 8,2% de los pacientes.

La mortalidad hasta los dos años de seguimiento se presentó en el 30,4% de los pacientes, estos datos son diferentes a los informados en un estudio multicéntrico en 51 países, que incluyó a 114 491 pacientes, donde reportan que la muerte ocurrió en el 45,8% de todos los pacientes ^19^. Otro estudio del Grupo Europeo de Trasplantes, que incluyó datos de 28 236 pacientes receptores de TPH informó que la mortalidad a los dos años de seguimiento fue del 38,2% ^20^. Si bien en comparación con otros estudios, la mortalidad que reportamos en nuestro estudio aparenta ser menor, esto se puede deber a múltiples factores que se deben considerar, como la alta heterogeneidad en la población, el tipo de quimioterapia recibida, el tiempo de seguimiento y el trasplante de donante no emparentado, esto limita la comparación de los resultados con otros estudios y muestra la clara necesidad de más información local.

La sepsis fue la principal causa de muerte a los 100 días de seguimiento pos-TPH. Si bien el riesgo de complicaciones infecciosas son una consecuencia de la inmunosupresión prolongada producida por la radio-quimioterapia antitumoral aplicada en el acondicionamiento previo al TPH ^1^. La muerte relacionada con complicaciones infecciosas ha disminuido con el paso de los años ^21^. Un estudio que analizó los datos de 114 491 pacientes pos-TPH entre 1980 y 2015, mostró que la mortalidad por infecciones bacterianas y fúngicas han disminuido con el paso de los años, aunque también informa que la frecuencia de muerte debido a infecciones de etiología viral o desconocida no cambió en el tiempo ^19^. La reducción en la mortalidad por complicaciones infecciosas se puede deber a una serie de estrategias que se han ido implementando con el paso del tiempo, como la profilaxis antibiótica, antifúngica y antiviral ^19^ y métodos de diagnóstico por reacción en cadena de la polimerasa ^21^. Si bien nuestro estudio no evaluó el impacto específico de estas intervenciones, su mención es relevante para contextualizar los esfuerzos globales para reducir complicaciones infecciosas.

A pesar de los avances realizados en trasplantes y la menor mortalidad global relacionada a las complicaciones, la recaída de enfermedad, tanto temprana (que ocurren entre 6 y 12 meses) como tardía, sigue siendo una complicación frecuente pos-TPH ^22^. Los tipos de cáncer más frecuentes asociados a la recaída pos-TPH son la leucemia mieloide aguda, el síndrome mielodisplásico, seguidos de leucemias linfoblásticas y linfomas ^23^. Esta probablemente sea la causa de porque en nuestro estudio, el grupo de pacientes con diagnóstico de leucemia mieloide aguda tuvo mayor riesgo de muerte a los dos años de seguimiento. La pérdida del efecto injerto contra tumor, por evasión inmunológica, se ha propuesto como un factor predisponente de esta complicación; otros factores son: los regímenes de acondicionamiento de baja intensidad, la edad avanzada de los pacientes y la inmunosupresión prolongada ^24^. Debido a que la recurrencia de la enfermedad se asocia a mayor mortalidad, resulta indispensable conocer qué factores (sobre todo aquellos modificables) se asocian al desarrollo de la recurrencia en nuestra población.

### Futuras investigaciones

Si bien, Europa y Norteamérica son las regiones del mundo con mayor cantidad de trasplantes por año, el número de procedimientos se ha incrementado en Latinoamérica ^2^^,^^3^. En Estados Unidos, en el año 2022 se realizaron aproximadamente 8500 trasplantes, de estos, más de 3500 fueron de donante no emparentado ^5^. El donante no emparentado, es aquella persona que, no siendo familiar directo, tiene compatibilidad HLA con el receptor. En Perú, el TPH de donante no emparentado aún no se realiza. Iniciar este tipo de trasplante podría incrementar el número de pacientes beneficiarios del procedimiento, lo que impactaría en su supervivencia.

Debido a la necesidad de más estudios en Perú, consideramos que estos podrían enfocarse en: 1) conocer la calidad de vida y complicaciones no hematológicas de los pacientes con sobrevida mayor a dos años; 2) evaluar qué factores se asocian al tiempo de espera entre la indicación del TPH hasta la realización del procedimiento; 3) identificar factores modificables que se asocian al desarrollo de complicaciones como la sepsis y la recurrencia de enfermedad; 4) replicar este primer estudio de manera prospectiva y multicéntrica para tener una idea más clara de las características del TPH en Perú y 5) realizar investigaciones que busquen conocer otros factores asociados a la mortalidad a los 5 y 10 años del TPH.

### Implicancias en la salud pública

Nuestros hallazgos evidencian desafíos sustanciales, pero también oportunidades clave para el sistema de salud peruano. Entre ellas destacan la necesidad de fortalecer las estrategias de prevención de infecciones. La alta incidencia de complicaciones infecciosas y la elevada mortalidad asociada a sepsis subrayan la urgencia de implementar protocolos robustos de vigilancia epidemiológica, especialmente enfocados en infecciones asociadas a la atención en salud y en la detección precoz de patógenos multirresistentes. En este contexto, el seguimiento clínico por médicos especialistas en infectología podría desempeñar un rol crucial en la reducción de estas complicaciones y en la mejora de los desenlaces clínicos.

La elevada mortalidad en receptores haploidénticos refuerza la necesidad de implementar un programa nacional de donantes no emparentados, como el modelo brasileño que incrementó la supervivencia en 22% ^3^, así como la necesidad de investigar más a fondo por qué ocurre la muerte en este grupo de pacientes. Actualmente, Perú no realiza este tipo de trasplantes, lo que limita las opciones para pacientes sin donantes familiares compatibles. Según un estudio reciente ^25^, la tasa media de TPH en América Latina es de 103 por cada 10 millones de habitantes (rango: 4-362); en Perú, la tasa es de 72, aún por debajo del promedio regional. Esta brecha podría atribuirse a la limitada infraestructura: solo existen diez centros acreditados para TPH en el Perú, siete de los cuales se concentran en la capital peruana y solo cuatro realizan trasplantes alogénicos ^11^^,^^26^^,^^27^^).^ La escasa disponibilidad de centros y su marcada centralización representan una barrera significativa para el acceso equitativo a este tratamiento.

Este estudio presenta algunas limitaciones que deben considerarse al interpretar los resultados. Primero, debido a la naturaleza observacional de nuestra investigación no pudimos determinar causalidad entre la asociación de variables. Segundo, si bien la mayoría de los pacientes que ingresan a TPH no suelen tener comorbilidades descompensadas, este dato no fue incluido, su ausencia en el análisis podría impedir ajustes por fragilidad basal y sobreestimar el efecto de otras variables. Tercero, dado que los resultados reportados son de solo un centro hospitalario, estos podrían no ser extrapolables a toda la población peruana, pero cabe mencionar que el HNERM es uno de los cuatro centros que realizan TPH alogénico en el Perú y es el que más trasplantes ha realizado. Cuarto, dado que realizamos un estudio retrospectivo, no pudimos analizar otros resultados de interés, como la calidad de vida y el desarrollo de enfermedades crónicas posterior al trasplante.

En conclusión, este estudio evidencia que la mortalidad post-TPH en el Perú (8,2% a 100 días y 30,4% a 2 años) es comparable a estándares internacionales, siendo la sepsis, el trasplante haploidéntico, la recaída de enfermedad y la edad >50 años los principales factores asociados. Los resultados justifican la implementación de protocolos estandarizados para prevención y manejo de infecciones por germenes multidrogo resistentes, programas de donantes no emparentados, e incremento del número de centros que realizan trasplante. La sostenibilidad de estos avances requerirá inversión en infraestructura, capacitación especializada y sistemas de seguimiento a largo plazo.
